# Hepatic alveolar echinococcosis: correlation between computed tomography morphology and inflammatory activity in positron emission tomography

**DOI:** 10.1038/s41598-020-68624-9

**Published:** 2020-07-16

**Authors:** Tilmann Graeter, Nina Eberhardt, Rong Shi, Julian Schmidberger, Ambros J. Beer, Meinrad Beer, Doris Henne-Bruns, Andreas Hillenbrand, Thomas F. E. Barth, Johannes Grimm, Wolfgang Kratzer, Beate Gruener

**Affiliations:** 1 Department of Diagnostic and Interventional Radiology, Ulm University Hospital, Albert-Einstein-Allee 23, 89081 Ulm, Germany; 2 Department of Nuclear Medicine, Ulm University Hospital, Albert-Einstein-Allee 23, 89081 Ulm, Germany; 3 Department of Internal Medicine I, Ulm University Hospital, Albert-Einstein-Allee 23, 89081 Ulm, Germany; 4 Department of General and Visceral Surgery, Ulm University Hospital, Albert-Einstein-Allee 23, 89081 Ulm, Germany; 5 Institute of Pathology, Ulm University, Albert-Einstein-Allee 23, 89081 Ulm, Germany; 6 Department of Internal Medicine III, Ulm University Hospital, Albert-Einstein-Allee 23, 89081 Ulm, Germany

**Keywords:** Radionuclide imaging, Computed tomography, Positron-emission tomography

## Abstract

Positron emission tomography-computed tomography (PET-CT) with 18F-fluorodesoxyglucose (FDG) is the imaging modality of choice for assessing inflammation surrounding hepatic alveolar echinococcosis (AE) lesions. This study is the first to evaluate FDG uptake in hepatic AE (n = 51) based on the standardized uptake value (SUV) and to correlate the SUVs with primary morphology and calcification patterns, based on the *Echinococcus multilocularis* Ulm Classification for Computed-Tomography (EMUC-CT). Our results show that the SUVs were increased for lesions with EMUC-CT types I-IV primary morphology, compared to the surrounding healthy liver tissue (SUV = 2.5 ± 0.4; p < 0.05). Type IV lesions included, by far, the highest number of PET-negative lesions. A comparison of lesions with different primary morphologies showed clear differences. The highest SUVs were found for types I and III, and the lowest was found for type IV. Type IV lesions (SUV, 3.8 ± 1.5) showed significantly lower uptake compared to type I (SUV, 6.9 ± 3.5; p = 0.030) and type III (SUV, 7.4 ± 3.9; p = 0.031) lesions. For type II lesions, the results showed only a statistical trend (SUV, 6.1 ± 3.1; p = 0.073). Due to the small number of cases, an evaluation of type V (n = 1) lesions was not possible. The different SUVs of lesions with different primary morphologies, particularly the lower FDG uptake observed in type IV lesions, suggested that these SUVs might reflect different stages of the disease.

## Introduction

### Prevalence

Alveolar echinococcosis (AE) is a dangerous zoonosis caused by the fox tapeworm, *Echinococcus multilocularis*. *E. multilocularis* is predominantly found in the cooler, temperate latitudes of the northern hemisphere^1,2^. Europe, particularly southern Germany, eastern France, northern Switzerland, and western Austria, are heavily populated with the parasite^1–3^. Outside central Europe, many human cases of AE have been found in China, particularly in the Tibetan plateau, and in Russia, particularly Siberia^1,2,4,5^. Most human cases of AE were reported in China^1,6–10^.

### General clinical picture and diagnosis

In over 98 percent, the liver is the organ most affected by AE. The larvae of *E*. *multilocularis* lead to the destruction and alteration of liver tissues, which could potentially foster malignant growth^12,13^. The diagnosis of AE often requires a combination of different imaging modalities, including ultrasonography (US), computed tomography (CT), magnetic resonance imaging (MRI), and 18F fluorodeoxyglucose-positron emission tomography (18F-FDG PET), and the results of immunodiagnostics (i.e., specific serology). Antibody testing is performed with a two-step approach; for sensitive *Echinococcus* species and *E. multilocularis*, an ELISA is performed, followed by a more specific Western blot^14,15^. When serology is inconclusive, a liver biopsy is warranted, with histopathology and polymerase chain reaction analyses to confirm the diagnosis^16^.

### Treatment of alveolar echinococcosis

An early diagnosis of AE is crucial. There is always an indication for treatment, due to the disease’s typically malignant character. Pharmacotherapy is used as the first-line treatment. In 1976, the prognosis improved considerably with the introduction of benzimidazoles (BMZ) for treatment^17^. Only about one-third of patients are diagnosed at a stage appropriate for primary local surgery. In some 70% of cases, a large proportion of the liver has become involved or there are extrahepatic manifestations, and thus, a curative resection is no longer possible. The primary aim is the complete (R0) resection of a lesion. However, even after a curative resection, patients should be monitored for possible recurrence for at least 10 years. BMZ therapy is predominantly parasitostatic and not parasiticidal. At present, there is no single parameter for assessing the vitality of hepatic foci.

### PET/CT examinations

PET/CT with FDG is an established tool for visualizing both the tumour burden and the inflammatory activity in different tissues. FDG-PET/CT is the current gold standard for assessing inflammation surrounding hepatic AE lesions, because it is the only imaging tool at present that can assess the disease-specific inflammatory activity of AE in the liver and extrahepatic lesions. Our research group first used PET examinations to assess hepatic lesions as far back as 1999^18^. Furthermore, in 2004, another study used PET to evaluate the effects of treatment discontinuation in a trial on albendazole^19^. PET is a highly sensitive method for detecting metabolic processes. To date, studies on AE with dedicated PET and hybrid PET/CT were mainly conducted to assess long-term therapy for patients with inoperable diseases^20^. Those studies typically determined disease activity in patients based on serological parameters^21^. More recent studies with PET and anti-*Echinococcus multilocularis* (EM)II/3-10 antibodies have shown that a negative serological status and negative PET findings could be achieved after long-term BMZ therapy^22^.

Various studies have compared PET and PET/CT results with other imaging modalities, such as ultrasound, colour doppler, contrast-enhanced ultrasound, and MRI with diffusion weighted imaging (DWI)^23–31^. However, even in 1999, when Reuter et al*.* conducted the first study to use PET in the diagnostic investigation of AE, the significance of calcification was recognized^18^. In earlier publications on AE, increased calcification in the liver was considered a sign of decreasing parasitic activity^32^, although increased calcification was also observed in lesions with persistent FDG uptake^19^.

### Classifications

**Figure 1:**
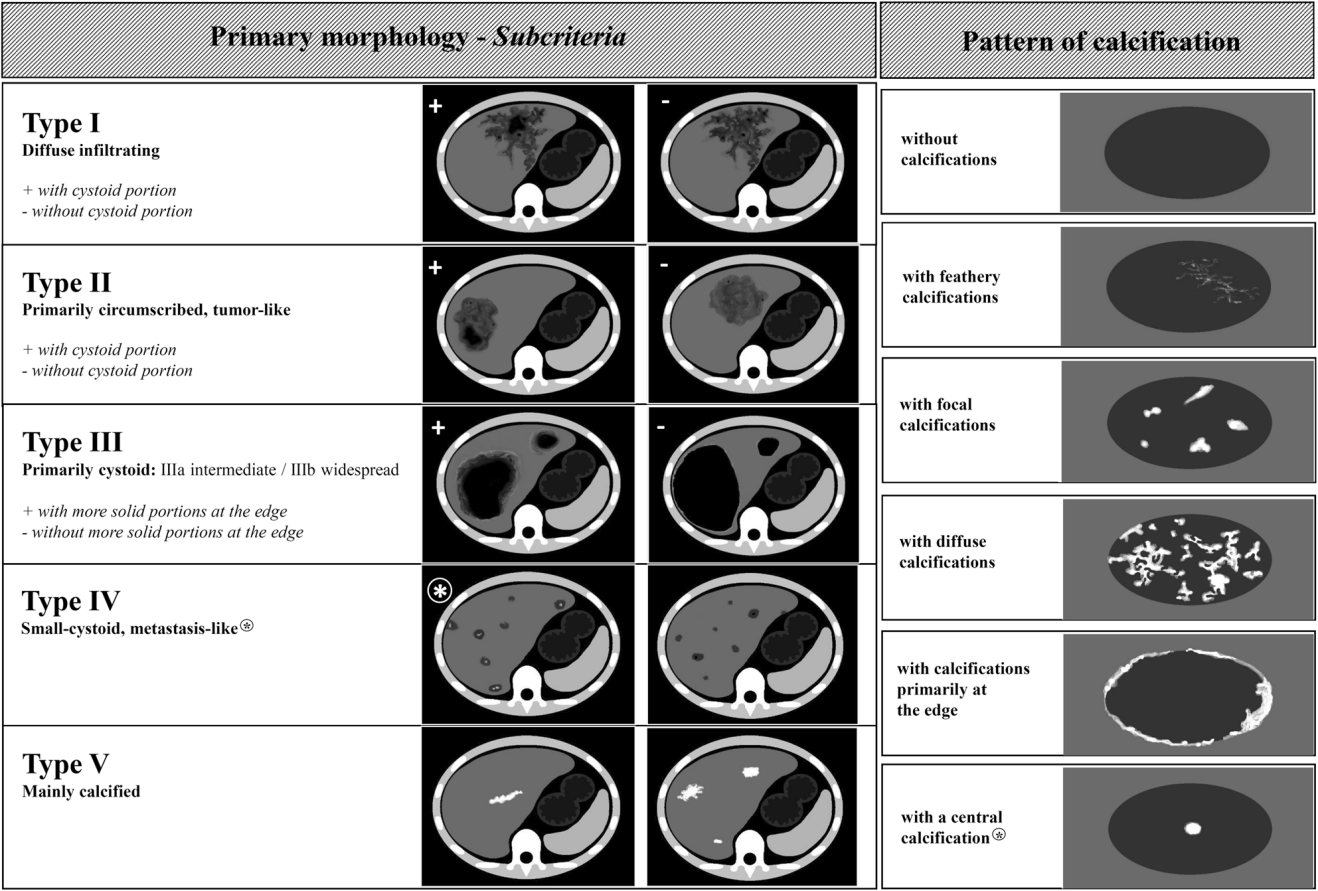
Overview of the EMUC-CT classification system, based on Graeter et al*. *(Left) Primary morphological types I–V and their subcriteria. (Right) Patterns of calcification. (EMUC-CT = *Echinococcus multilocularis* Ulm Classification for Computed Tomography). The two columns of classification are primarily considered separately; then, in principal, they can be freely combined. There are two exceptions: (1) The calcification pattern called “with a central calcification*” can only occur in lesions with type IV primary morphology, and (2) primary morphology type V is not associated with a pattern of calcification.

At present, AE classifications are available for MRI, CT, and ultrasound data^,^^33,34^. The *E. multilocularis* Ulm classification for computed tomography (EMUC-CT), developed by Graeter et al*.*, was the first to provide a precise description of different types of liver lesions and calcification patterns, based on CT-defined morphology. These descriptions improved the diagnostic recognition of AE, in view of its complex morphology, and allowed a more precise description of the changes observed with disease progression (Fig. 1). The potential value of different CT morphological criteria for assessing AE inflammatory activity has not been evaluated previously. Based on the Kodama classification for MRI data, a comparative study between PET/CT and MRI showed that the presence of microcysts was associated with a significant increase in the metabolic activity detected with PET/CT^28,33^. Recent studies that compared contrast-enhanced ultrasonography with 18F-FDG PET/CT have shown a good correlation between FDG uptake and perilesional vascularization^26,30,31^. In cystic echinococcosis (CE), morphological findings with imaging corresponded to disease activity and could be used to manage treatment^35^. We hypothesized that it might also be possible to capture morphological images that reflect the AE disease process.

The present study aimed to evaluate PET/CT activity, measured as the standardized uptake value (SUV), and evaluate its relationship to the primary morphological type and calcification pattern of hepatic AE lesions, defined with the EMUC-CT classification system. In particular, we investigated whether the different primary morphological types and calcification patterns of AE were associated with different levels of inflammatory activity.

## Methods

### Ethical statement

This study was approved by the local Ethics Committee of the University of Ulm, and it was conducted in accordance with the Declaration of Helsinki (ref. No. 440/15). At the time they were included in the database, patients had provided written informed consent to use the collected image data for future retrospective analyses. The study did not include patients under 18 years of age. All data were analysed anonymously.

### Patients

We retrospectively retrieved data on patients with hepatic AE from the German National Echinococcus database at Ulm University Hospital. All patients had been treated at a dedicated echinococcosis outpatient clinic during 2001–2017 and had undergone an 18-FDG PET/CT examination (n = 193).

### Inclusion and exclusion criteria

Of the 193 identified cases, we excluded patients that had undergone surgery for echinococcal lesions (n = 26) or had only undergone pre-therapeutic PET/CT imaging, without a serology measurement (n = 36). We also excluded all patients that had previously received BMZ therapy (n = 80). We included only patients diagnosed with ‘confirmed’ or ‘probable’ AE, according to the WHO case definition^35^. PET/CT examinations obtained at places other than in our clinic were not taken into consideration. The final cohort for this retrospective study included 51 treatment-naive patients with hepatic AE.

### 18-FDG PET/CT examinations and SUV measurements

The 18-FDG PET/CT examinations were performed with the PET/CT scanner from General Electrics (GE) Discovery LS (until February 2011) or with the PET/CT scanner, Biograph mCT-S (40), from Siemens. The former scanner was run in 2D PET mode, and the latter scanner measured PET in full 3D mode. Moreover, the Siemens scanner permitted time of flight (TOF) and point spread function corrections. For both scanners, the measuring time per PET bed position was 2.5 min.

CT images were viewed at 1.5–5.0 mm slice thicknesses, with reconstruction intervals equal to or less than the slice thickness. Quantitative PET images were viewed at a 5-mm slice thickness (colour scale), with a pixel size of 4.25 mm (GE scanner) or 4.07 mm (Siemens scanner). PET images were overlaid onto CT images of the same slice thickness (grey scale) with a Siemens Syngo® Via workstation (software version VB30). The examined patients received F-18-FDG applications of approximately 320 to 340 MBq, and the measurements were performed after an incubation period of around 60–90 min. CT was done in shallow expiration after careful instruction of the patients.

The 18-FDG PET/CT examination data were classified according to the primary AE lesion morphology and the pattern of calcification, based on the EMUC-CT^11^ (Fig. 1). The portal venous phase (n = 45; 88.2%) and the non-contrast dataset (n = 6; 11.8%) were used to evaluate the lesions. When a patient had more than one hepatic AE lesion, the largest lesion was considered in each case.

The classification of AE lesions was performed by two specialist trainee radiologists with different experience levels (about 3 years and 6 years). They independently assessed multiplanar reconstructions of 4-mm or 5-mm slices stored in the Picture Archiving and Communication System (PACS) program.

**Figure 2:**
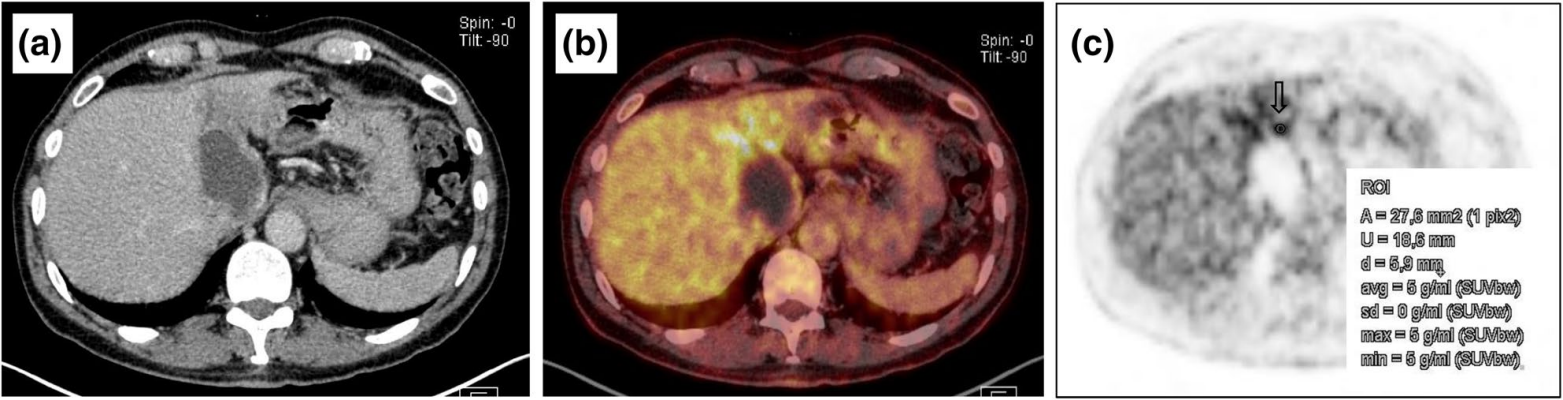
18-FDG PET/CT examination and measurement of SUV in an active hepatic alveolar echinococcosis lesion (EMUC-CT type I). (**a**) Contrast enhanced CT scan in the venous phase, (**b**) combined PET/CT, (**c**) quantitative measurement of the maximum SUV in a PET scan, for a defined ROI (arrow).

**Figure 3:**
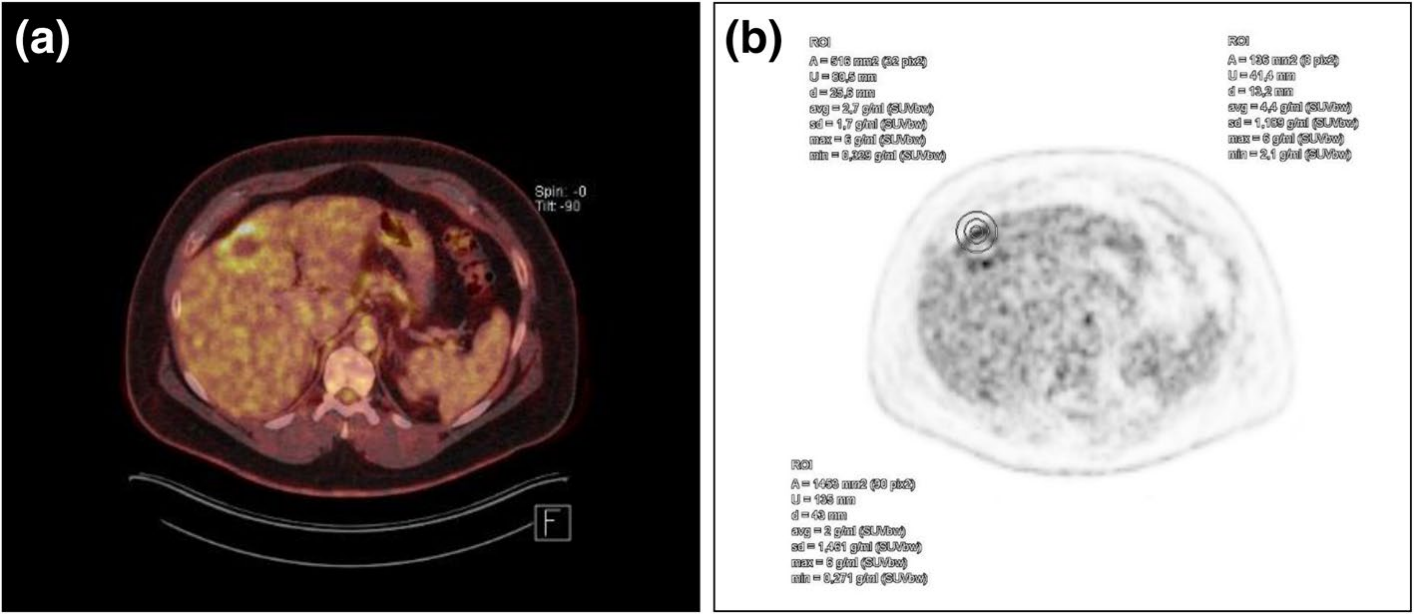
SUVmax is independent of the ROI size, and the measurement is not subject to a partial volume effect, because it is a measurement of punctate signals within the ROI. (**a**) An active hepatic alveolar echinococcosis lesion (EMUC-CT type II), (**b**) the same SUVmax was measured for different ROI sizes (indicated with concentric circles).

In PET images, the SUVs of the AE lesions that did stand out visually from the background liver tissue (PET-positive lesions) were measured as the maximum SUV (SUVmax). The area for measurement was first selected visually, based on the maximum detectable activity, and then, that area was measured as a region of interest (ROI) with the SUV-measuring tool in the PACS program (Fig. 2). The SUVmax was independent of the ROI size, and the measurement was not subject to a partial volume effect, because it measured punctate signals within the ROI (Fig. 3).

To compare uptake in the PET-positive AE lesions to the background activity of normal liver, additional measurements from representative areas of the liver (e.g., without vessels) were carried out with ROIs to determine the SUVmean and SUVmax of the normal liver parenchyma. The ROIs for liver background were determined in three consecutive PET slices, within a diameter of 2–3 cm, with a Siemens Syngo® Via workstation (software version VB30). We calculated the mean values of these measurements, and we evaluated the ratios of the SUV for the lesion to the mean SUV for normal liver background. Lesions that did not stand out visually from the background liver tissue were referred to as PET-negative lesions. No SUV measurements were performed for PET-negative lesions.

### Statistical analysis

Statistical analyses were performed with SAS, Version 9.4 (SAS Institute Inc. Cary, NC, USA). The data were first analysed descriptively to determine the frequency, median, minimum, and maximum, and we calculated the mean and standard deviation. The Shapiro–Wilk test was used to check for normal distributions. We used the Wilcoxon rank sum test to determine differences between variables without a normal distribution. The Kruskal–Wallis test was used to determine differences between groups. Pearson's chi-squared and the exact Fisher test were used to evaluate differences in frequency between two variables. For correlations, we used the Pearson correlation coefficient. P-values < 0.05 were interpreted as statistically significant with a probability of error of five percent.s

## Results

This cohort study included 34 women (66.7%) and 17 men (33.3%) with hepatic AE. The mean age of the patients was 55.6 ± 17.0 years. Six patients (11.8%) were in the 18–30 age group, 23 (45.1%) were in the 31–60 age group, and 22 (43.1%) were over 60 years old.

### Classification according to the EMUC-CT

The AE cases were classified by two specialist trainee radiologists, according to the EMUC-CT, with an inter-rater reliability of 92%. The lesion types were distributed as follows: 17 (33.3%) were type I, 11 (21.6%) were type II, nine (17.7%) were type III a/b, 13 (25.5%) were type IV, and only one patient (1.96%) had a type V lesion (Table 1). The largest echinococcosis-related lesion was found most often in the right lobe of the liver (N = 31 patients, 60.8%), but it was located in the left lobe in 14 patients (27.5%), and both lobes were involved in six patients (11.8%).

**Table 1:** Characteristics of patients in the cohort investigated (n = 51).

No previous treatment (n = 51)	N (%)	Mean ± SD
**Gender**		
Male Female	17 (33.3) 34 (66.7)	
**Age (mean ± SD)**		55.6 ± 17.0
18–30 years 31–60 years > 60 years	6 (11.8) 23 (45.1) 22 (43.1)	
**EMUC-CT primary morphology type**		**Size of the lesion (mm)**
Type I Type II Type III Type IV Type V	17 (33.3) 11 (21.6) 9 (17.7) 13 (25.5) 1 (2.0)	77.2 ± 27.3 62.4 ± 27.3 87.9 ± 50.1 22.6 ± 15.9 32.0 ± 0.0
**EMUC-CT calcification pattern**		
Without calcification Feathery calcification Diffuse calcification Focal calcification Calcification primarily at the edge Central calcification No calcification pattern	16 (32.0) 6 (12.0) 11 (22.0) 13 (26.0) 2 (4.0) 2 (4.0) Primary morphological type IV	
**Site of the space-occupying lesion in the liver**		
right lobe left lobe both lobes	31 (60.8) 14 (27.4) 6 (11.8)	
**EMUC-CT primary morphology types for** **PET-negative lesions (n = 10)**		
Type I Type II Type III Type IV Type V	1/17 (5.9%) 0/11 (0.0%) 1/9 (11.1%) 7/13 (53.8%) 1/1 (100.0%)	

According to the EMUC-CT, no calcification was found in 16 (32.0%) patients. Six patients (12.0%) showed feathery calcifications, 11 (22.0%) had diffuse calcifications, and 13 (26.0%) had focal calcifications. Two patients (4.0%) had the rarest pattern of calcification, primarily at the edges of the liver, and another two patients (4.0%) had a central calcification. In accordance with the authors’ algorithm, EMUC-CT type V was not allocated a calcification pattern.

### SUVs in relation to EMUC-CT primary morphology and calcification patterns

#### Primary morphology

**Figure 4:**
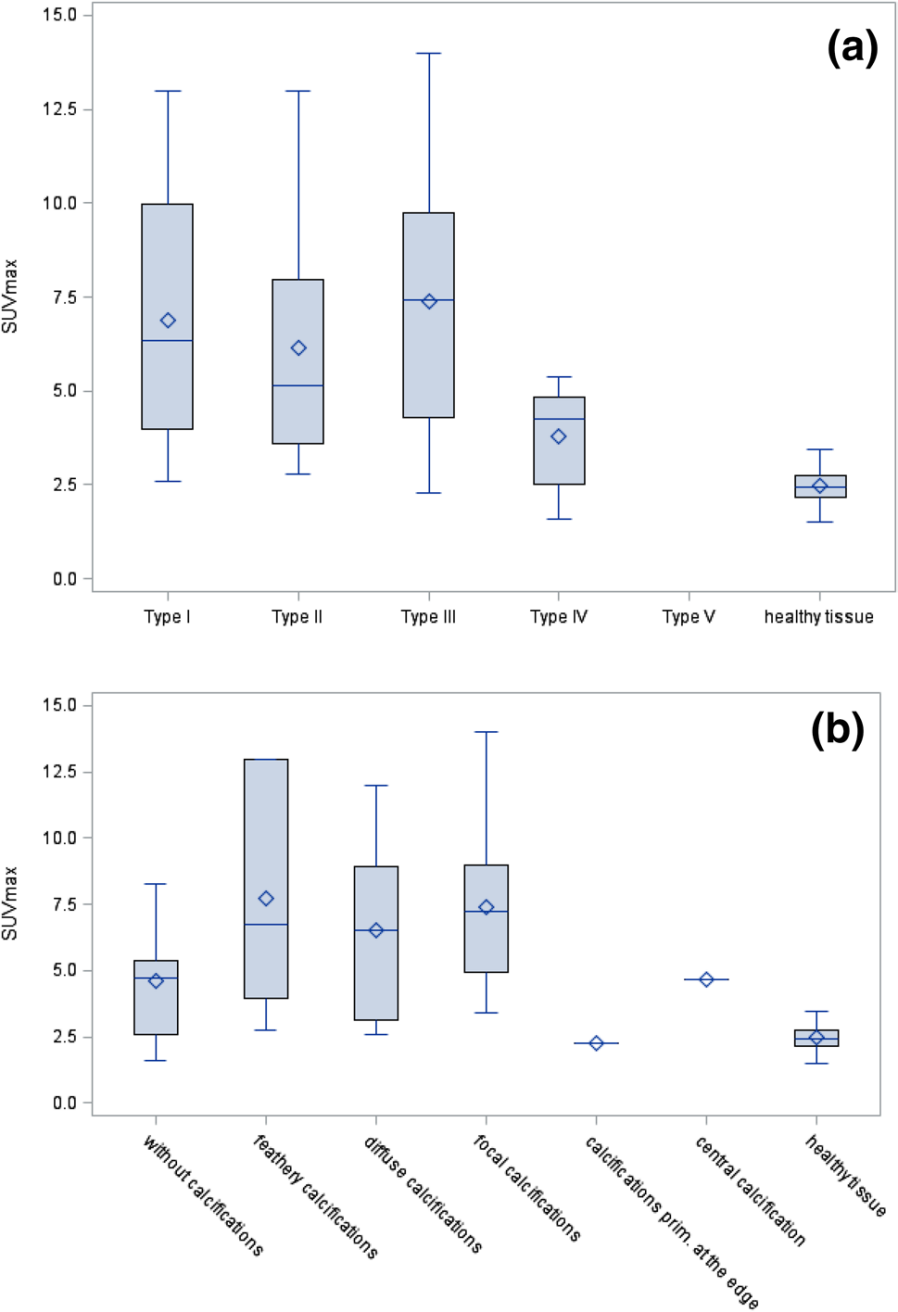
SUV measurements according to the primary morphology and the calcification pattern. (**a**) Box plot shows SUVs according to primary morphological types I-V, compared to the SUV in healthy tissues. (**b**) Box plot shows SUVs according to calcification patterns, compared to the SUV in healthy tissues.

There were significant differences in SUVs between the various primary morphologies and the healthy liver tissue (χ2 = 50.3; p < 001). The SUVs of PET-positive lesions (n = 41) were significantly different from reference SUVs in healthy liver tissues (SUV, 2.5 ± 0.4; n = 51; Table 2) in type I (6.9 ± 3.5; p < 0.001), type II (6.1 ± 3.1; p < 0.001), type III (7.4 ± 3.9; p < 0.001), and type IV (3.8 ± 1.5; p = 0.032) lesions. Because type IV lesions showed remarkably lower FDG uptake than the other lesion types, we focused on type IV lesions. According to the primary morphological type, we found statistically significant differences between the SUV of type IV lesions (3.8 ± 1.5) and the SUVs of type I (6.9 ± 3.5; p = 0.030) and type III (7.4 ± 3.9; p = 0.031) lesions. However, type IV and type II SUVs were not significantly different (3.8 ± 1.5 vs. 6.1 ± 3.1; p = 0.073). Moreover, types I, II, and III lesions showed no significant differences in SUVs (Table 2, Fig. 4a). Due to the small number of type V lesions, we could not calculate the significance level.

**Table 2:** SUVs of PET-positive lesions, according to the EMUC-CT primary morphological type (n = 41).

Primary morphology type	SUVs in AE lesions, mean ± SD	SUVs healthy liver tissue, mean ± SD (n = 51)	p-value
Type I (n = 16)	6.9 ± 3.5	2.5 ± 0.4	< .001
Type II (n = 11)	6.1 ± 3.1	2.5 ± 0.4	< .001
Type III (n = 8)	7.4 ± 3.9	2.5 ± 0.4	0.001
Type IV (n = 6)	3.8 ± 1.5	2.5 ± 0.4	0.032
Type V (n = 0)	–	2.5 ± 0.4	–
	**Mean ± SD**	**Type IV (n = 6)**	**p-value**
Type I (n = 16)	6.9 ± 3.5	3.8 ± 1.5	0.030
Type II (n = 11)	6.1 ± 3.1	3.8 ± 1.5	0.073
Type III (n = 9)	7.4 ± 3.9	3.8 ± 1.5	0.031
Type V (n = 0)	–	3.8 ± 1.5	–
	**Mean ± SD**	**Type III (n = 8)**	**p-value**
Type I (n = 16)	6.9 ± 3.5	7.4 ± 3.9	0.415
Type II (n = 11)	6.1 ± 3.1	7.4 ± 3.9	0.241
	**Mean ± SD**	**Type II (n = 11)**	**p-value**
Type I (n = 16)	6.9 ± 3.5	6.1 ± 3.1	0.374

Statistical significance was set at p < 0.05.

N = 10 lesions turned out to be PET-negative. The PET-negative lesions for each morphological type are shown separately in Table 1. By far, most PET negative lesions were observed in type IV lesions (7 out of 13 cases, 53.8%).

#### Calcification pattern

There were significant differences in SUVs between the various calcification patterns and the healthy liver tissue (χ2 = 54.0; p < 001). Compared to the reference SUV in healthy liver tissues (SUV, 2.5 ± 0.4; n = 51), we found significantly different SUVs in lesions without calcifications (4.6 ± 2.1; p < 001), with feathery calcifications (7.7 ± 4.7; p < 001), with diffuse calcifications (6.5 ± 3.3; p < 001), and with focal calcifications (7.4 ± 3.3; p = 0.032; Table 3). Furthermore, the central and scatter tendencies of SUVs are shown in Table 3, according to the calcification pattern. The highest SUVs were observed in lesions with feathery calcifications (7.7 ± 4.7). In particular, we observed significantly different SUVs between lesions without calcifications and lesions with focal calcifications (4.6 ± 2.2 vs. 7.4 ± 3.3; p = 0.022). There were no other differences between the groups with and without calcifications (Table 3, Fig. 4b).

**Table 3:** SUVs of PET-positive lesions, according to the EMUC-CT calcification pattern (n = 41).

EMUC-CT calcification pattern	SUVs in AE lesions, mean ± SD	SUVs in healthy liver tissue, mean ± SD (n = 51)	p-value
Without calcification (n = 10)	4.6 ± 2.1	2.5 ± 0.4	< .001
Feathery calcification (n = 6)	7.7 ± 4.7	2.5 ± 0.4	< .001
Diffuse calcification (n = 11)	6.5 ± 3.3	2.5 ± 0.4	< .001
Focal calcification (n = 12)	7.4 ± 3.3	2.5 ± 0.4	< .001
Calcification primarily at the edge (n = 1)	2.3 ± 0.0	2.5 ± 0.4	–
Central calcification (n = 1)	4.7 ± 0.0	2.5 ± 0.4	–
	**Mean ± SD**	**Mean SUV for lesions without calcification (n = 10)**	**p-value**
Feathery calcification (n = 6)	7.7 ± 4.7	4.6 ± 2.1	0.127
Diffuse calcification (n = 11)	6.5 ± 3.3	4.6 ± 2.1	0.085
Focal calcification (n = 12)	7.4 ± 3.3	4.6 ± 2.1	0.022
Calcification primarily at the edge (n = 1)	2.3 ± 0.0	4.6 ± 2.1	–
Central calcification (n = 1)	4.7 ± 0.0	4.6 ± 2.1	–
	**Mean ± SD**	**Mean SUV for lesions with feathery calcification (n = 6)**	**p-value**
Diffuse calcification (n = 11)	6.5 ± 3.3	7.7 ± 4.7	0.241
Focal calcification (n = 12)	7.4 ± 3.3	7.7 ± 4.7	0.481
Calcification primarily at the edge (n = 1)	2.3 ± 0.0	7.7 ± 4.7	–
Central calcification (n = 1)	4.7 ± 0.0	7.7 ± 4.7	–
	**Mean ± SD**	**Mean SUV for lesions with diffuse calcification (n = 11)**	**p-value**
Focal calcification (n = 12)	7.4 ± 3.3	6.5 ± 3.3	0.249
Calcification primarily at the edge (n = 1)	2.3 ± 0.0	6.5 ± 3.3	–
Central calcification (n = 1)	4.7 ± 0.0	6.5 ± 3.3	–
	**Mean ± SD**	**Mean SUV for lesions with focal calcification (n = 12)**	**p-value**
Calcification primarily at the edge (n = 1)	2.3 ± 0.0	7.4 ± 3.3	–
Central calcification (n = 1)	4.7 ± 0.0	7.4 ± 3.3	–
	**Mean ± SD**	**Mean SUV for lesions with calcification primarily at the edge (n = 1)**	**p-value**
Central calcification (n = 1)	4.7 ± 0.0	2.3 ± 0.0	–

Statistical significance was set at p < 0.05.

If, in view of its comparatively lower SUVs, one just considers the collective of lesions without calcifications (n = 16) dichotomously with regard to their FDG-PET positivity/negativity in general, it becomes apparent that in this group all lesions which were PET-negative (n = 6) belonged to type IV.

Five other lesions without calcifications (PET-positive lesions) also had type IV morphology and showed lower SUVs (3.6 ± 1.6) compared to the overall collection. In contrast, the other lesions without calcifications (n = 5) had comparatively higher SUVs: type I (4.7 ± 0.2), type II (7.6 ± 0.0), and type III (5.4 ± 4.0).

### Serology according to the primary morphology type

#### Echinococcus multilocularis

Serum samples were positive for *E*. *multilocularis* (Em2 +) in 15/17 (88.2%) lesions with a type I primary morphology. Em2 + samples were associated with 9/11 (81.8%) type II lesions and 6/9 (66.7%) type III lesions (Table 4). In contrast, type IV lesions were associated with serum samples that were negative for Em2 + in 11/13 (84.6%) cases. The positive/negative serological Em2 + ratio differed significantly in Type IV lesions compared to type I (p < 0.001), type II (p = 0.002), and type III (p = 0.022) lesions. There were also significant differences in the SUVs of lesions associated with Em2 + negative and Em2 + positive serology (3.8 ± 1.6 vs. 7.2 ± 3.4; p = 0.003).

**Table 4:** Serology, according to the EMUC-CT primary morphological type (n = 51).

Morphology	Detection of *E*. *multilocularis* (Em2 +) in serum, n (%)			Indirect haemagglutination test (IHA) results, n (%)			Level of IgE in serum (IU/ml)	
	Em2 + negative	Em2 + positive	p-value	IHA negative	IHA positive	p-value	Mean ± SD	p-value
Type I	2 (11.8%)	15 (88.2%)	< .001	5 (29.4%)	12 (70.6%)	< .001	1166.0 ± 2550.3	0.014
Type II	2 (18.2%)	9 (81.8%)	0.002	6 (54.6%)	5 (45. 5%)	0.011	737.1 ± 1699.8	0.093
Type III	3 (33.3%)	6 (66.7%)	0.022	3 (33.3%)	6 (66. 7%)	0.001	618.8 ± 900.1	0.316
Type IV	11 (84.6%)	2 (15.4%)	Reference	13 (100.0%)	0 (0.0%)	Reference	150.2 ± 291.3	Reference
Type V	1 (100.0%)	0 (0.0%)	0.857	1 (100.0%)	0 (0.0%)	< .001	–	–

Statistical significance was set at p < 0.05.

#### Indirect haemagglutination test

The indirect haemagglutination test (IHA) results were positive in 12/17 (70.6%) patients with type I lesions, in 5/11 (45.5%) patients with type II lesions, and 6/9 (66.7%) patients with type III lesions (Table 4). In particular, type IV lesions were associated with a negative IHA in all cases (13/13; 100.0%). Therefore, the positive/negative IHA serological findings associated with type IV lesions differed significantly from the findings associated with type I (p < 0.001), type II (p = 0.011), and type III (p = 0.001) lesions. There were no significant differences in SUVs between lesions associated with IHA-negative and IHA-positive findings (6.1 ± 3.5 vs. 6.5 ± 3.3; p = 0.328).

#### Immunoglobulin E (IgE):

Type IV lesions also had significantly different IgE serology (150.2 ± 291.3 IU/ml), compared to type I (1166.0 ± 2550.3 IU/ml; p = 0.014), type II (737.1 ± 1699.8 IU/ml; p = 0.093), and type III (618.78 ± 900.1 IU/ml; p = 0.316) lesions (Table 4). We found a correlation between serum IgE levels and lesion SUVs (r = 0.33; p = 0.035).

## Discussion

Hybrid PET/CT has long been the gold standard for assessing inflammatory activity surrounding AE lesions in the liver, both at the initial diagnosis and during the course of treatment^18–22,36^. The present study was the first to quantify FDG uptake in hepatic AE and to relate the SUV to EMUC-CT-defined primary morphology and calcification patterns^11^. The most important result regarding the primary morphology was that PET-positive type IV lesions showed significantly lower FDG uptake than types I and III lesions. Furthermore, among the various lesion types, type IV lesions were, by far, most frequently PET-negative. The most important result regarding the calcification patterns was the lower FDG uptake in lesions without calcifications compared to lesions with calcifications. Moreover, this effect appeared to be associated with type IV morphology.

Earlier studies of FDG uptake in hepatic AE lesions did not include any differentiation among the different morphological types, based on a CT classification. Furthermore, most previous studies did not calculate the SUVs. A study by Ehrhardt et al*.* was the first to quantify FDG uptake and compare it with contrast-enhanced ultrasound findings^25^. Studies by Kaltenbach et al*.* and Li et al*.* also made quantitative comparisons between FDG uptake and contrast-enhanced ultrasonography in AE lesions^26,30^. Moreover, Azizi et al*.* conducted an important study, where they evaluated the PET activity in AE lesions and correlated the results with the Kodama classification of lesions based on MRI^28^. They showed that PET positivity was linked to the presence of microcysts. However, the Kodama classification of lesions based on MRI, which exclusively used T2-weighted images (T2w), did not include any class equivalent to the type IV lesion defined in the EMUC-CT system ^33^. In cases where the central alveolus is very small, small lesions (like type IV lesions) are sometimes undetected with MRI or misinterpreted as simple cysts, due to the poor resolution of T2w images, which makes it difficult to delimit solid necrotic border areas. Considering the special features of type IV lesions revealed in our study, the lack of this class in the Kodama classification system limits its ability to make an overall assessment of the disease. Therefore, the Kodama classification should be revised to include the small EMUC-CT type IV lesion.

**Figure 5:**
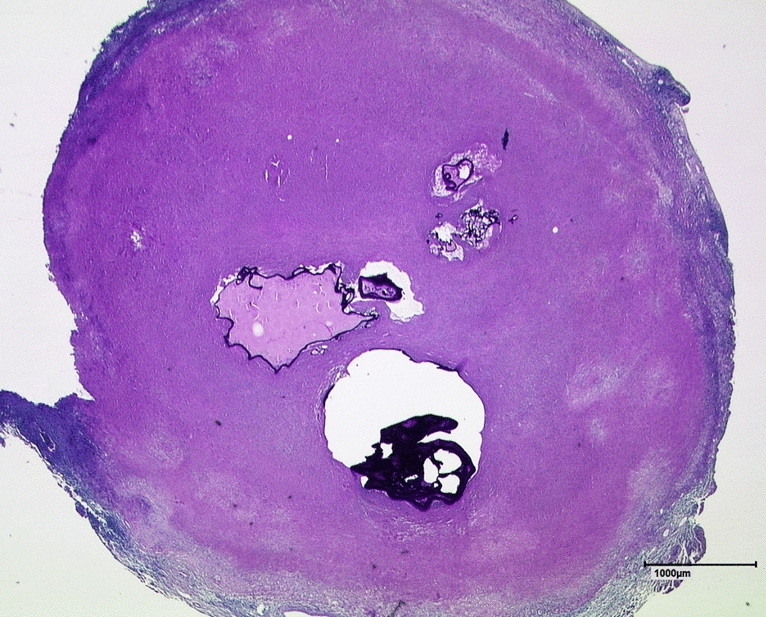
Histology of a EMUC-CT type IV AE lesion with central alveoli. This liver section was stained with PAS and is shown at 12.5 × magnification. Note the wide surrounding area of solid necrosis. (AE = alveolar echinococcosis, PAS = periodic acid-Schiff).

The frequent PET negativity of type IV lesions and the lower FDG uptake of PET-positive type IV lesions, compared to other lesion types, might be attributable to the structure of type IV lesions (Fig. 5). Type IV lesions have a central lamellar body and viable alveoli (or the central area becomes calcified over time), which are surrounded by an occasionally wide margin of solid necrosis. This necrotic margin makes the centre inaccessible to the blood supply necessary for FDG uptake. Findings from recent studies have demonstrated that the small type IV lesions represent initial lesions that might either become worse or undergo involution^37^. Presumably, the human body has the ability to initially isolate the active lesion centre with the surrounding necrosis and, in some cases, this isolation can be sustained for long periods. In contrast, types I, II, and III lesions showed higher SUVs than type IV lesions. Consequently, we might assume that these lesion types represent different stages of AE that are more advanced than the initial stages represented by type IV lesions. In these more advanced stages, the disease activity occurs at the edges of the lesions, which allows the lesions to extend further ^37^. Because the presence of type I, II, or III lesions appears to indicate an aggravated stage of the disease, we do not expect any significant difference in the FDG uptake among these lesion types. Accordingly, types I, II, and III lesions showed hardly any PET-negative results, compared to type IV lesions. The only primary morphology that did not show any positive FDG uptake was type V morphology. Only one of type V lesion was found in our study; however, we speculated that this type of lesion must be a nonviable, residual manifestation of AE.

Although MRI provides better detection of microcysts above a certain size, CT is clearly superior for resolving the outer edges of lesions and demonstrating calcification in AE^23^. Mueller et al*.* demonstrated the presence of macrocalcification in AE lesions, based on MRI performed with susceptibility-weighted imaging (SWI)^38^. However, further differentiation of the calcification pattern was not possible with MRI, particularly for small areas of calcification. A study by Reuter et al*.*^18^ used PET to describe microcalcification and macrocalcification in AE lesions. Analogous to other inflammatory diseases, it is reasonable to assume that microcalcification might be more closely associated with increases in inflammatory activity, compared to macrocalcification. In addition to describing the primary morphological patterns, the EMUC-CT offers careful differentiation of the different calcification features in AE lesions. These calcification patterns were not primarily defined to indicate different inflammatory activity, but rather, to provide greater detail of the complex morphological appearance of these lesions. The aim of including calcification patterns was to improve the diagnosis and the ability to monitor changes in disease progression^11^. However, in the present study, we also investigated a potential correlation between FDG uptake and the calcification component of the classification.

In our analysis of calcification patterns, we found notably lower FDG uptake in lesions with calcifications primarily at the edges, lesions with a central calcification, and lesions without calcifications, compared to lesions with other calcification patterns. We found significantly lower SUVs in lesions without calcifications than in lesions with focal calcifications. Among the calcification patterns with higher SUVs, the more discreet patterns, like feathery calcifications and focal calcifications, showed the highest FDG uptakes. Indeed, these patterns showed even higher SUVs than the more dominant pattern of diffuse calcification. The highest SUVs were observed in lesions with feathery calcifications.

When we only considered the subgroup of lesions without calcifications, we found that, within this group, all PET-negative lesions were type IV. Furthermore, the PET-positive, non-calcified type IV lesions showed lower SUVs compared to types I, II, and III lesions without calcifications. Therefore, the association between the lack of calcification and reduced FDG uptake might be attributed to the type IV morphology. The central calcification pattern, which only occurred in EMUC-CT type IV lesions, had relatively lower SUVs than lesions with other calcification patterns. This finding also provided evidence for the effect of type IV morphology.

A recent study by Brumpt et al. qualitatively examined PET activity in AE lesions classified solely by the presence of micro- and macrocalcifications^39^. Consistent with our finding that the highest SUVs were observed in lesions with feathery calcifications, Brumpt et al. demonstrated a relationship between PET positivity and microcalcifications, but they only examined dichotomous groups. The more differentiated calcification classes, particularly lesions without calcifications, and the various, complex underlying lesion morphologies, were not considered in the Brumpt et al. study. Our data showed that, for a comprehensive assessment of the relationship between FDG uptake and the different types of AE lesions, it is important to combine both aspects of lesions: the primary morphology and the calcification pattern.

Finally, the present study also highlighted a particular feature of type IV lesions: their relationship to serology. Type IV lesions were frequently associated with the negative detection of Em2 + , IHA, and IgE in serum. This serology was significantly different from the serology associated with types I, II, and III lesions. Together with the results on AE serology in a recent study by Gottstein et al*.*^40^, this result again supported the hypothesis that type IV might be an initial lesion that could either become worse or undergo involution^37^. The special features of type IV lesions might directly influence the planning of pharmacotherapy and surgical treatment, particularly in determining whether, or for how long, treatment might be relevant.

Based on studies that used PET/CT as the gold standard, it is likely that intermodal comparative studies might also be of interest in the future. Indeed, the value of PET/MRI in the diagnostic investigation of AE cannot be adequately assessed at the present time^41^.

This study had some limitations. The small numbers of cases for some of the individual calcification patterns prevented the analysis of how FDG uptake was related to the calcification patterns. Nevertheless, in addition to detecting some significant differences between groups, our results also showed some trends in the relationships between calcification patterns and FDG uptake. Another potential limitation was that the records only noted the dominant calcification pattern of each lesion. It is possible that a more differentiated examination might provide more advanced results. For example, different calcification patterns simultaneously present in one lesion and their precise location might be related to the maximum FDG uptake. Every effort should be made to carry out large-scale studies to address these questions.

Concerning PET data acquisition a minor limitation may be motion artefacts. In the routine scan protocol of the nuclear medicine department involved, patients are carefully instructed to hold their breath in shallow expiration for the CT part with satisfactory results for the analysis. Nevertheless, for future prospective studies it could be useful to work with respiratory gating or data-driven gating with continuous bed motion in order to prevent motion artefacts and related false positive or negative measured values of the PET^42,43^.

While the present study focused on PET activity and morphology of a single AE lesion, future PET-based studies could also focus on the global disease burden of AE. PET-based global disease assessment is increasingly used in various clinical areas such as inflammatory disorders, Alzheimer’s disease, atherosclerosis, liver diseases and in malignancies^44–48^. Oncological global disease scores are based on parameters like total metabolic tumour volume or total lesion glycolysis. Comparable study approaches could also contribute to a global assessment in the course of disseminated AE diseases. Furthermore, in PET studies based on the global disease assessment, potential technical confounding factors such as respiratory motion or partial volume effects would not have a negative impact on the result^48^. However, it must be taken into account that PET activity in alveolar echinococcosis merely reflects the surrounding inflammatory reaction and cannot always be equated with the actual vitality of the parasite.

In contrast to a global disease assessment, the focus of the current study was intentionally just set on the respective biggest liver lesion and not on all lesions together. Accordingly, the aim of the present study was to look at characteristics of the PET activity for different types and calcifications of hepatic AE lesions in CT which might help to improve the statements about the activity or even the stage of various lesions also from the morphological point of view.

## Conclusions

Hybrid PET/CT has long been the gold standard for assessing inflammatory activity surrounding AE lesions in the liver, both at the initial diagnosis and during the course of treatment. The present study was the first to quantify FDG uptake with SUV in different hepatic AE lesion types, described by EMUC-CT definitions of primary morphology and calcification patterns^11^. The most important result regarding the primary morphology was that PET-positive type IV lesions showed significantly lower FDG uptake than types I and III lesions. Furthermore, among the various lesion types, type IV lesions were, by far, most frequently PET-negative. The most important result regarding the calcification patterns was the lower FDG uptake in lesions without calcifications compared to lesions with calcifications. Moreover, this effect appeared to be associated with type IV morphology. The highest SUVs obtained overall were in lesions with feathery calcifications. This study showed that, for a comprehensive assessment of the relationships between FDG uptake and different types of AE lesions, it is important to combine both aspects of the lesion: the primary morphology and the calcification pattern.

The small, type IV lesions primarily represent initial lesions that could either progress or undergo involution. Presumably, the human body has the ability to isolate the active centre with the surrounding necrosis, initially; in some cases, this isolation can be sustained for long periods. Based on our findings that types I, II, and III lesions showed higher SUVs than type IV lesions, we might conclude that these lesion types represent different stages of more advanced AE lesions, where the disease activity is located at the edges; thus, these lesions can extend further over time. This study also highlighted a particular feature of type IV lesions, based on serology. Type IV lesions were frequently associated with negative serology for Em2 + , IHA, and IgE. This serology differed significantly from the serology associated with types I, II, and III lesions. Furthermore, the FDG uptake differed significantly between cases with positive and negative Em2 + serology. We found a correlation between the levels of Em2 + and IgE in serum.

The special features of type IV lesions might directly influence the planning of pharmacotherapy and surgical treatments, particularly in determining whether treatment is relevant.

## Data availability

The datasets used and analysed in the current study are available from the corresponding author upon reasonable request.

## References

[1] Baumann S (2019). Worldwide literature on epidemiology of human alveolar echinococcosis: a systematic review of research published in the twenty-first century. Infection..

[2] Moro P, Schantz PM (2009). Echinococcosis: a review. Int. J. Infect. Dis..

[3] Romig T (1999). An epidemiologic survey of human alveolar echinococcosis in southwestern Germany. Römerstein Study Group. Am. J. Trop. Med. Hyg..

[4] Torgerson PR, Keller K, Magnotta M, Ragland N (2010). The global burden of alveolar echinococcosis. PLoS. Negl. Trop. Dis..

[5] Deplazes P (2017). Global distribution of alveolar and cystic echinococcosis. Adv. Parasitol..

[6] Craig PS (1992). A large focus of alveolar echinococcosis in central China. Lancet.

[7] Craig PS (2003). Echinococcus multilocularis. Curr. Opin. Infect. Dis..

[8] Zhang W (2015). Epidemiology and control of echinococcosis in central Asia, with particular reference to the People’s Republic of China. Acta. Trop..

[9] Craig PS (2006). Epidemiology of human alveolar echinococcosis in China. Parasitol. Int..

[10] Tiaoying L (2005). Echinococcosis in Tibetan populations, western Sichuan Province, China. Emerg. Infect. Dis..

[11] Graeter T (2016). Proposal of a computed tomography classification for hepatic alveolar echinococcosis. World J. Gastroenterol..

[12] Ammann RW, Eckert J (1996). Cestodes. Echinococcus. Gastroenterol. Clin. North. Am..

[13] Eckert J, Deplazes P (2004). Biological, epidemiological, and clinical aspects of echinococcosis, a zoonosis of increasing concern. Clin. Microbiol. Rev..

[14] Eckert J, Gemmell MA, Meslin FX, Pawlowski ZS (2001). WHO-OIE Manual on Echinococcosis in Humans and Animals: A Public Health Problem of Global Concern.

[15] Stojkovic M, Mickan C, Weber TF, Junghanss T (2015). Pitfalls in diagnosis and treatment of alveolar echinococcosis: a sentinel case series. BMJ Open Gastroenterol..

[16] Kern P (2010). Clinical features and treatment of alveolar echinococcosis. Curr. Opin. Infect. Dis..

[17] Torgerson PR (2008). Alveolar echinococcosis: from a deadly disease to a well-controlled infection. Relative survival and economic analysis in Switzerland over the last 35 years. J. Hepatol..

[18] Reuter S (1999). Pericystic metabolic activity in alveolar echinococcosis: assessment and follow-up by positron emission tomography. Clin. Infect. Dis..

[19] Reuter S (2004). Structured treatment interruption in patients with alveolar echinococcosis. Hepatology.

[20] Reuter S (2008). Long-term follow-up of metabolic activity in human alveolar echinococcosis using FDG-PET. Nuklearmedizin..

[21] Stumpe KD (2007). F-18-fluorodeoxyglucose (FDG) positron-emission tomography of Echinococcus multilocularis liver lesions: prospective evaluation of its value for diagnosis and follow-up during benzimidazole therapy. Infection.

[22] Ammann RW (2015). Outcome after discontinuing long-term benzimidazole treatment in 11 patients with non-resectable alveolar echinococcosis with negative FDG-PET/CT and anti-EmII/3-10 serology. PLoS Negl. Trop. Dis..

[23] Reuter S (2001). Alveolar liver echinococcosis: a comparative study of three imaging techniques. Infection.

[24] Coşkun A (2004). Alveolar echinococcosis of the liver: correlative color Doppler US, CT, and MRI study. Acta Radiol..

[25] Ehrhardt AR (2007). Assessment of disease activity in alveolar echinococcosis: a comparison of contrast enhanced ultrasound, three-phase helical CT and [(18)F] fluorodeoxyglucose positron emission tomography. Abdom. Imaging..

[26] Kaltenbach TE (2015). Determination of vitality of liver lesions by alveolar echinococcosis. Comparison of parametric contrast enhanced ultrasound (SonoVue®) with quantified 18F-FDG-PET CT. Nuklearmedizin..

[27] Li H (2015). Efficiency of liposomal albendazole for the treatment of the patients with complex alveolar echinococcosis: a comparative analysis of CEUS, CT, and PET/CT. Parasitol. Res..

[28] Azizi A (2015). Alveolar echinococcosis: correlation between hepatic MRI findings and FDG-PET/CT metabolic activity. Abdom. Imaging..

[29] Zheng J, Wang J, Zhao J, Meng X (2018). Diffusion-weighted MRI for the initial viability evaluation of parasites in hepatic alveolar echinococcosis: comparison with positron emission tomography. Korean J. Radiol..

[30] Li J, Dong J, Yang L, Li X, Song T (2018). Comparison of [^18^F]fluorodeoxyglucose positron emission tomography and contrast-enhanced ultrasound for evaluation of hepatic alveolar echinococcosis activity. Ultrasound Med. Biol..

[31] Schwarze V (2018). The use of contrast-enhanced ultrasound (CEUS) for the diagnostic evaluation of hepatic echinococcosis. Clin. Hemorheol. Microcirc..

[32] Ammann RW, Fleiner-Hoffmann A, Grimm F, Eckert J (1998). Long-term mebendazole therapy may be parasitocidal in alveolar echinococcosis. J. Hepatol..

[33] Kodama Y (2003). Alveolar echinococcosis: MR findings in the liver. Radiology.

[34] Kratzer W (2015). Proposal of an ultrasonographic classification for hepatic alveolar echinococcosis: Echinococcosis multilocularis Ulm classification-ultrasound. World J. Gastroenterol..

[35] Brunetti E, Kern P, Vuitton DA (2010). Writing Panel for the WHO-IWGE. Expert consensus for the diagnosis and treatment of cystic and alveolar echinococcosis in humans. Acta Trop..

[36] Bresson-Hadni S (2006). Imaging aspects and non-surgical interventional treatment in human alveolar echinococcosis. Parasitol. Int..

[37] Grimm, J., *et al.* Combined analysis of computed tomography and histology leads to an evolutionary concept of *Echinococcus multilocularis* infections in humans. *PLoS Negl Trop Dis. *(submitted) (2019)

[38] Mueller J, Stojkovic M, Kauczor HU, Junghanss T, Weber TF (2018). Performance of magnetic resonance susceptibility-weighted imaging for detection calcifications in patients with hepatic echinococcosis. J. Comput. Assist. Tomogr..

[39] Brumpt E (2019). AE hepatic lesions: correlation between calcifications at CT and FDG-PET/CT metabolic activity. Infection.

[40] Gottstein, B., *et al*. Diagnostic and follow-up performance of serological tests for different forms/courses of alveolar echinococcosis. *Food and Waterborne Parasitology*. e00055 (2019). 10.1016/j.fawpar.2019.e00055 PMC7034017 32095626

[41] Lötsch F (2017). FDG-PET/MRI in alveolar echinococcosis. Int. J. Infect. Dis..

[42] Büther F, Jones J, Seifert R, Stegger L, Schleyer P, Schäfers M (2020). Clinical evaluation of a data-driven respiratory gating algorithm for whole-body positron emission tomography with continuous bed motion. J Nucl Med.

[43] Smeets EMM (2019). Optimal respiratory-gated [18F]FDG PET/CT significantly impacts the quantification of metabolic parameters and their correlation with overall survival in patients with pancreatic ductal adenocarcinoma. EJNMMI Res..

[44] Høilund-Carlsen PF, Edenbrandt L, Alavi A (2019). Global disease score (GDS) is the name of the game!. Eur. J. Nucl. Med. Mol. Imaging..

[45] Saboury B, Salavati A, Werner T, Alavi A (2013). Integrative imaging biomarker: Global disease assessment with PET/CT, a combination of functional and structural image quantification. J. Nucl. Med..

[46] Marin-Oyaga VA (2015). Feasibility and performance of an adaptive contrast-oriented FDG PET/CT quantification technique for global disease assessment of malignant pleural mesothelioma and a brief review of the literature. Hell J. Nucl. Med..

[47] Lee SJ (2019). Development and validation of an 18 F-fluorodeoxyglucose-positron emission tomography with computed tomography-based tool for the evaluation of joint counts and disease activity in patients with rheumatoid arthritis. Arthritis Rheumatol..

[48] Raynor, WY., *et al.* The necessity of global disease assessment in optimal management of patients with cancer. *Elsevier Health Sciences.* Part II, 140–145 (2018). In Alavi, A., Salavati, A., Gholamrezanezhad, A., Guermazi, A. (eds)* PET-CT-MRI Applications in Musculoskeletal Disorders, Part II, An Issue of PET Clinics*.

